# Supplementary material for: Dynamic transcriptomic profiles of zebrafish gills in response to zinc supplementation
Source: BMC Genomics. 2010 Oct 11;11:553. doi: 10.1186/1471-2164-11-553 (PMC3091702; doi:10.1186/1471-2164-11-553)
Supplement: Additional file 2 — Interactive Direct Interaction Network representing the molecular interactions between zinc, copper, iron, calcium and proteins encoded by transcripts changed by zinc supplementation. Mini web-site containing index.html and hyperlinked pages in subdirectory describing a Direct Interaction Network automatically generated based on curated interactions contained within the proprietary PathwayArchitect database. Ovals represent proteins and the circles symbolize metal ions. Objects are coloured by their abundance in zebrafish at the time-point they were significantly different from the control is a scale from -4 fold (dark green) to +4 fold (dark red). Where significant differences were found at more than one time-point, the colour overlay shows expression at the first instance. Dark blue squares denote 'binding', and light blue squares 'expression'; green squares stand for 'regulation', green diamonds for 'metabolism', and green circles for 'promoter binding'. Arrow heads indicate directionality of the interaction where annotated. All nodes and edges can be further interrogated by selecting the relative area of the image. [file 1471-2164-11-553-S2.zip › PathwayArchitect Zn xs DIN/82109.html]

# PROTEIN: DRAP1

|  |  |
| --- | --- |
| Name | DRAP1 |
| Type | PROTEIN |
| Description | DR1-associated protein 1 (negative cofactor 2 alpha) |
| Note | Transcriptional repression is a general mechanism for regulating transcriptional initiation in organisms ranging from yeast to humans. Accurate initiation of transcription from eukaryotic protein-encoding genes requires the assembly of a large multiprotein complex consisting of RNA polymerase II and general transcription factors such as TFIIA, TFIIB, and TFIID. DR1 is a repressor that interacts with the TATA-binding protein (TBP) of TFIID and prevents the formation of an active transcription complex by precluding the entry of TFIIA and/or TFIIB into the preinitiation complex. The protein encoded by this gene is a corepressor of transcription that interacts with DR1 to enhance DR1-mediated repression. The interaction between this corepressor and DR1 is required for corepressor function and appears to stabilize the TBP-DR1-DNA complex. |
| Alias | Negative co- factor 2 alpha |
|  | DRAP1 |
|  | Dr1-associated protein 1 |
|  | DR1-associated corepressor |
|  | 2310074H19Rik |
|  | NC2-alpha |
|  | DR1-associated protein 1 |
|  | Drap1 |
|  | NC2 alpha |
|  | negative cofactor 2 alpha |


---

|  |  |
| --- | --- |
| GO Component | nucleus |


---

|  |  |
| --- | --- |
| GO ID | GO:0005634 |
|  | GO:0003677 |
|  | GO:0003714 |
|  | GO:0006355 |
|  | GO:0000122 |
|  | GO:0005515 |
|  | GO:0006350 |
|  | GO:0008134 |
|  | GO:0003700 |


---

|  |  |
| --- | --- |
| MIM | MIM:602289 |


---

|  |  |
| --- | --- |
| Connectivity | 27 |


---

|  |  |
| --- | --- |
| Entrez ID | 66556 |
|  | 10589 |


---

|  |  |
| --- | --- |
| Agilent ID | A\_23\_P138857 |
|  | A\_23\_P138856 |
|  | A\_14\_P103875 |
|  | A\_51\_P479929 |
|  | A\_51\_P479933 |
|  | A\_53\_P164602 |
|  | A\_24\_P107277 |


---

|  |  |
| --- | --- |
| Cellular Localization | Nucleus |
|  | Organelle |
|  | Cell |


---

|  |  |
| --- | --- |
| Pathway | Zn xs inventory |
|  | Zn xs DIN |


---

|  |  |
| --- | --- |
| GO Process | negative regulation of transcription from RNA polymerase II promoter |
|  | regulation of transcription, DNA-dependent |
|  | transcription |


---

|  |  |
| --- | --- |
| UniGene | Mm.275446 |
|  | Hs.356742 |
|  | Hs.381222 |


---

|  |  |
| --- | --- |
| Affymetrix Probeset ID | 1451676\_at |
|  | 203258\_at |
|  | 230160\_x\_at |
|  | 39076\_s\_at |
|  | 96617\_at |
|  | Hs.135102.0.A1\_3p\_at |
|  | X96506\_s\_at |
|  | g5453635\_3p\_at |
|  | TC26653\_s\_at |
|  | TC26654\_f\_at |
|  | TC26655\_f\_at |


---

|  |  |
| --- | --- |
| GO Function | protein binding |
|  | transcription factor activity |
|  | DNA binding |
|  | transcription corepressor activity |
|  | transcription factor binding |


---

|  |  |
| --- | --- |
| Nucleotide | AK154251 |
|  | CT010290 |
|  | BC010025 |
|  | AK010152 |
|  | BG719065 |
|  | AK005445 |
|  | NM\_024176 |
|  | NM\_006442 |
|  | X96506 |
|  | AK087456 |
|  | AI685498 |
|  | BC002090 |


---

|  |  |
| --- | --- |
| Protein | Q9D6N5 |
|  | CAA65358 |
|  | BAE32465 |
|  | NP\_006433 |
|  | AAH10025 |
|  | CAJ18498 |
|  | Q14919 |
|  | AAH02090 |
|  | NP\_077138 |
|  | BAC39882 |
|  | BAB26737 |


---

|  |  |
| --- | --- |
| Organism | Mammal |


---

|  |  |
| --- | --- |
| Location | chromosome 11, 11q13.3 (Homo sapiens) |
|  | chromosome 19, 19 A (Mus musculus) |


---

|  |  |
| --- | --- |
